# Soybean cyst nematode culture collections and field populations from North Carolina and Missouri reveal high incidences of infection by viruses

**DOI:** 10.1371/journal.pone.0171514

**Published:** 2017-01-31

**Authors:** Casey L. Ruark, Stephen R. Koenning, Eric L. Davis, Charles H. Opperman, Steven A. Lommel, Melissa G. Mitchum, Tim L. Sit

**Affiliations:** 1 Department of Entomology and Plant Pathology, North Carolina State University, 2510 Thomas Hall, Raleigh, North Carolina, United States of America; 2 Division of Plant Sciences and Bond Life Sciences Center, University of Missouri, 371H Bond Life Sciences Center, Columbia, Missouri, United States of America; University of California, Riverside, UNITED STATES

## Abstract

Five viruses were previously discovered infecting soybean cyst nematodes (SCN; *Heterodera glycines*) from greenhouse cultures maintained in Illinois. In this study, the five viruses [ScNV, ScPV, ScRV, ScTV, and SbCNV-5] were detected within SCN greenhouse and field populations from North Carolina (NC) and Missouri (MO). The prevalence and titers of viruses in SCN from 43 greenhouse cultures and 25 field populations were analyzed using qRT-PCR. Viral titers within SCN greenhouse cultures were similar throughout juvenile development, and the presence of viral anti-genomic RNAs within egg, second-stage juvenile (J2), and pooled J3 and J4 stages suggests active viral replication within the nematode. Viruses were found at similar or lower levels within field populations of SCN compared with greenhouse cultures of North Carolina populations. Five greenhouse cultures harbored all five known viruses whereas in most populations a mixture of fewer viruses was detected. In contrast, three greenhouse cultures of similar descent to one another did not possess any detectable viruses and primarily differed in location of the cultures (NC versus MO). Several of these SCN viruses were also detected in *Heterodera trifolii* (clover cyst) and *Heterodera schachtii* (beet cyst), but not the other cyst, root-knot, or reniform nematode species tested. Viruses were not detected within soybean host plant tissue. If nematode infection with viruses is truly more common than first considered, the potential influence on nematode biology, pathogenicity, ecology, and control warrants continued investigation.

## Introduction

Until recently, plant parasitic nematodes (PPN) of the genera *Longidorus*, *Trichodorus*, and *Xiphinema* were the only nematodes known to harbor viruses, but these genera serve as vectors of plant viruses and replication does not occur within the worm [1,2,3]. Few studies have attempted to demonstrate viral replication in nematodes. An early study observed that root knot nematode (*Meloidogyne incognita*) was infected with a filterable, virus-like pathogen, but the infective agent was never identified [4]. The 1970s brought a collection of electron microscopy images of virus-like particles in nematodes; however, insufficient definitive information left the scientific community doubtful [5,6,7]. More recently, *Caenorhabditis elegans* were experimentally infected with the insect-infecting *Flock house virus* (FHV), and successful replication within the nematode was demonstrated [8]. Natural infection by Orsay virus in *C*. *elegans* and Santeuil virus in *C*. *briggsae* were subsequently discovered by high throughput sequencing [9]. Le Blanc virus infection was also discovered in *C*. *briggsae* [10] shortly afterwards. Another study localized Orsay, Santeuil, and Le Blanc viruses primarily to a small number of intestinal cells of *C*. *elegans* and *C*. *briggsae* [11]. The effect of these viruses on *Caenorhabditis* species is yet to be determined.

The first viruses confirmed to infect plant-parasitic nematodes were discovered by serendipity within the soybean cyst nematode (SCN; *Heterodera glycines*) via transcriptome pyrosequencing [12]. SCN is the leading pathogen of soybean in the United States [13]. The four viruses initially discovered in SCN possess negative-sense, single-stranded RNA genomes [12]. An additional virus, SCN virus-5 (SbCNV-5), was later discovered [14] which has a positive-sense, single-stranded RNA genome. The five viruses were detected within TN10, an inbred SCN line from single-cyst descent, and several other greenhouse populations of SCN that also originated from single-cyst descent [15]. SbCNV-5 was the only virus also documented within field samples of SCN in Illinois [14]. Thus far, the negative-sense RNA viruses have not been reported within field populations of SCN.

The objective of the investigation here was to assess if virus infection of SCN is more widespread than originally demonstrated [12,14]. A large-scale survey was conducted for SCN to determine if virus infection could be detected and to assess the relative levels of potential SCN viral infections. Both field and greenhouse populations were examined to deduce whether viruses infect nematodes directly in agricultural systems and are not artifacts of heavily inbred greenhouse populations. The study focused on the known viruses in SCN to confirm previous reports [12,14] as well as additional nematode species that have not yet been investigated for virus infection. Plant host tissue was tested to determine if viruses could potentially infect host plants as well as nematodes. A critical component of this study was to determine if viruses are actively replicating in SCN and if these viruses are detectable in multiple nematode life stages. As SCN is the predominant pathogen of soybean, further knowledge of nematode viruses could prove useful in understanding nematode ecology and implementing successful management strategies.

## Materials and Methods

### Sample collection

Total RNA was extracted from 0.1 g of tissue from the leaves, stems, roots, and pods of 1 to 3 month-old soybean plants non-infected with SCN in a greenhouse at North Carolina State University (NCSU). Soybean cultivars tested include Bedford, Hartwig, Jake, NC Raleigh, NC Roy, and Williams 82. Additionally, a split root trial of NC Roy was repeated twice where half the root system was infected with either LY1 (infected with five known viruses) or MM21 (no known viral infection). Roots tested for viruses were selected from the non-infected half of the root system to avoid possible contamination of nematodes. In addition to roots, leaves, stems, and pods were also sampled for SCN viral content. ELF1B was used as a measure of soybean plant levels via qRT-PCR, and SCN internal controls GAPDH and 18*S* were tested to further rule out contamination via nematodes (Table 1). The backgrounds of inbred SCN populations used in this study are documented in Table 2. Field SCN populations were collected from infested soybean fields in North Carolina (NC) and Missouri (MO); multiple soil cores were collected and pooled from each field. Distributions of SCN in NC have been documented by [16] and in MO by [17]. NC samples were propagated in the greenhouse for several months on roots of susceptible soybean cultivars, and field samples of SCN from MO were tested directly in the assays below without further propagation of the populations. SCN cysts were extracted from soil via water flotation and collection on nested sieves of screen sizes 20 (850 μm) and 60 (250 μm). SCN eggs were extracted by crushing cysts with a Tenbroeck homogenizer and further separated from soil debris by centrifugation in 70% sucrose and collection on a size 500 screen (25 μm). To minimize contamination, extraction equipment was bleached and/or autoclaved between SCN populations.

**Table 1:** Primers used for amplifying selected regions of virus RNA-dependent RNA polymerases (RdRPs) from total RNA samples.

Primer	Sequence [5’ to 3’]	Source
ELF1B-F ^a^	GTTGAAAAGCCAGGGGACA	[20]
ELF1B-R ^a^	TCTTACCCCTTGAGCGTGG	
ScNV-R ^a^	ACGAGCAGCGGATGAGTTTAA	[12]
ScNV-RU ^a^^,^^b^	GGTCCTCCGCTGCCCTATGGTGGCCACTTCCTGTGCTTCT	
ScPV-R ^a^	CAGTGGGACGGCAAAATCTT	
ScPV-RU ^a^^,^^b^	GGTCCTCCGCTGCCCTATGGCAGTGGGACGGCAAAATCTT	
ScRV-R ^a^	TTGACAAGTGCGGGTTTGAG	
ScRV-RU ^a^^,^^b^	GGTCCTCCGCTGCCCTATGGGTCCCCCTGCCCCATTATC	
ScTV-R ^a^	AGCCCCAAGGACCTGGTTT	
ScTV-RU ^a^^,^^b^	GGTCCTCCGCTGCCCTATGGCTTCCGGTGTAGCAGGGAGAT	
SbCNV-5-F ^a^	GGAACTGTGTCGCGGTTTG	[14]
SbCNV-5-R ^a^^,^^b^	TGCACTGAGGCATTTCACAAC	
HgFAR1-F ^a^	CCATTTGCCGCCTTTGGA	
HgFAR1-5-R ^a^	GGGATCAATTCGCGGTATTCG	
GAPDH-F ^a^	TCCAAGGCATAGAAAGACGACG	Designed for this study
GAPDH-R ^a^	AACAAGTCATTGGACGGCATCA	
ScNV-F500 ^b^	ACATAGTCAGTGGCCGATTC	
ScPV-F500 ^b^	CTCGTCGAAGAGCTGTGTTAC	
ScRV-F500 ^b^	GCGACTGTTCTCGCTCCTGA	
ScTV-F500 ^b^	TGTGTTTTACATGCCGGCCTC	
SbCNV-5-F500 ^b^	CTTGTTCGAGGTGAGTGTGG	
ScNVQ-F ^a^	GGTCCTGCTTAGCTTGTGA	
ScNVQ-R ^a^	CATCTGTGGTGATTGGAC	
ScPVQ-F ^a^	CGCAAGATGGAAGACCAG	
ScPVQ-R ^a^	GGTGATGGTGGAGAATTTAG	
ScRVQ-F ^a^	GGCATACCCGTCGGCAAC	
ScRVQ-R ^a^	GTCCAACCGGGACACAGCC	
ScTVQ-F ^a^	CTCATGTCACCGTCTCTGTGC	
ScTVQ-R ^a^	GCAGTTACGCAAGACTGGTCTAG	
SbCNV5Q-F ^a^	CATGACAGCAAAGTGTGCCG	
SbCNV5Q-R ^a^	CGGTCCAACTTCGCGCCAC	
PPN18S-F ^a^	GGTAGTGACGAGAAATAACG	
PPN18S-R ^a^	CTGCTGGCACCAGACTTG	

RNAs were extracted from *Glycine max* (internal control ELF1B), *Heterodera glycines* (internal controls HgFAR1, GAPDH and PPN18*S*) or other nematode species (internal control PPN18*S*).

^a^ Used for qRT-PCR detection.

^b^ PCR amplification of approximately 500 bp segment of RdRP.

**Table 2:** Inbreeding protocol, date of origin and HG type of SCN greenhouse populations used in this study.

Population	Inbreeding protocol	Origin date	HG type at time of sample collection	Citation
LY1	Synthetic isolate, selected for reproduction on ‘Hartwig’ by Lawrence Young.	1998	1–7	[15]
LY2	Field isolate from Tennessee mass selected on ‘Hartwig’ by Lawrence Young.	2000	1–7	
MM1	PA3 mass-selected on susceptible Essex x Forrest 63 (EXF63) recombinant inbred line	2006	0	M. Mitchum, unpubl.
MM2	PA3 mass-selected on resistant Essex x Forrest 67 (EXF67) recombinant inbred line	2006	1.2.3.5.6.7	
MM3	PA3 mass-selected on susceptible Evans x PI 209332 near-isogenic line 7923S	2006	2.5.7	
MM4	PA3 mass-selected on resistant Evans x PI 209332 near-isogenic line 7923R	2006	2.5.7	
MM7	PA3 x TN20 outselected on Peking	2006	1.2.3.5.6.7	[26]
MM8	PA3 x TN20 outselected on PI 88788	2006	2.5.7	
MM10	PA3 x TN20 outselected on PI 437654	2006	1–7	
MM16	Mass-selection of cysts recovered from a Mississippi, MO field population on PI 437654.	2013	1–7	M. Mitchum, unpubl.
MM18	Mississippi County, MO field population HG 1.2.3.5.6.7 (Race 4) subjected to rotation on susceptible soybean cvs. Lee74, Essex, Macon and Williams 82.	2013	1.2.3.5.6.7	
MM19	Mississippi County, MO field population HG 1.2.3.5.6.7 (Race 4) subjected to rotation on soybeans with PI 88788 type resistance.	2013	1.2.3.5.6.7	
MM21	Mississippi County, MO field population HG 1.2.3.5.6.7 (Race 4) subjected to rotation on soybeans with Hartwig type resistance.	2013	1.2.3.-	
MM23	Mississippi County, MO field population HG 1.2.3.5.6.7 (Race 4) subjected to rotation on soybeans with PI 88788, Peking and Hartwig type resistance.	2013	1.2.3.-	
MM24	Mississippi County, MO field population HG 1.2.3.5.6.7 (Race 4) subjected to rotation on soybeans with PI 88788, Peking and Hartwig type resistance and susceptible soybeans.	2013	1.2.3.-	
OP20^a^	Field isolate from North Carolina selected by single cyst decent on PI 88788.	-	1.2.3.5.6.7	[25]
OP25^a^	Field isolate from North Carolina selected by single cyst decent on Lee 68.	-	0	
OP50^a^	Field isolate from North Carolina selected by single cyst decent on PI 90763.	-	1.2.3.5.6.7	
PA3	Prakash Arelli “Race 3” maintained on Williams 82.	-	0	[15]
TN1	Terry Niblack, Gene pool isolate originally prepared by V. Dropkin maintained on Macon soybean.	1980	1.2.3.5.6.7	
TN2/tomato	Isolate from a potato field inbred by mass selection on Tiny Tim tomato.	1990	0	
TN2/soybean	TN2 inbred by mass selection on soybean Williams 82.	1990	0	
TN6	TN1 selected by single-cyst descent on Peking.	1996	1.2.3.5.6.7	
TN7	TN1 selected by single-cyst descent on PI 88788.	1996	2.5.7	
TN8	TN1 selected by single-cyst descent on PI 90763.	1996	1.3.6.7	
TN12	TN1 inbred by mass selection on Pickett.	1988	1.2.3.5.6.7	
TN13	TN1 inbred by mass selection on Peking.	1988	1.3.5.6.7	
TN14	TN1 inbred by mass selection on PI 88788.	1988	1.2.5.7	
TN15	TN1 inbred by mass selection on PI 90763.	1988	1–7	
TN19	LY1 selected by single-cyst descent on Hartwig.	1988	1–7	
TN20	LY1 selected by single-cyst descent on PI 437654.	1999	1–7	
TN21	Field isolate from Missouri mass selected on Hartwig.	2001	1–7	
TN22	TN2 mass selected on PI 88788.	1999	1.2.5.7	
VL1	Virgil Leudders ‘Hg1’ (high on 89008, low on 209332); mass selected on PI 89008.	1985	2.5.7	

Unless otherwise noted, populations were maintained at University of Missouri (MU) greenhouses. LY = Lawrence Young; MM = Melissa Mitchum; OP = Charlie Opperman; PA = Prakash Arelli; TN = Terry Niblack; VL = Virgil Leudders.

^a ^Maintained in both NCSU and MU greenhouses.

### RNA extraction and cDNA synthesis

RNA was extracted from approximately 10,000 eggs of SCN greenhouse cultures as well as MO field samples and 5 to 10 cysts of NC field populations. Samples were prepared for total RNA extraction by homogenization with three 3-mm glass beads in a 1.5 ml tube on a Silamat S6 (Ivoclar Vivadent, Amherst, NY). Alterations in sample preparation were made for cysts in which a motorized pestle was first used to improve sample destruction. When extracting total RNA from plant tissue, a different modification was made as it was flash frozen in liquid nitrogen immediately before bead beating. The samples were then processed for total RNA using TRIzol^®^ Reagent (Invitrogen, Carlsbad, CA) under the guidelines of the manufacturer’s protocol adapted from [18]. Total RNA concentrations were analyzed via Nanodrop 1000 (Thermo Fisher Scientific, Waltham, MA). cDNA was synthesized by incubating approximately 1 μg RNA with 0.06 μg random primers (Invitrogen) for 10 minutes at 70°C followed by rapid cooling on ice. Next, 4 μl GeneAmp^®^ 10X PCR Buffer II (Applied Biosystems, Foster City, CA), 5.5 mM MgCl_2_, 0.5 μM deoxynucleotide solution mix, 32 U Murine RNase Inhibitor (New England BioLabs, Ipswich, MA), and 50 U Multiscribe^™^ Reverse Transcriptase (Applied Biosystems) were added before additional incubations of 42°C and 70°C for 15 minutes each.

### Virus detection via qRT-PCR

NCBI GenBank accession numbers for SCN viral sequences are HM849038 (ScNV), HM849039 (ScRV), HM849040 (ScPV), HM849041 (ScTV) and KF726084 (SbCNV-5). All primers used in this research were synthesized by Eurofins Genomics (Huntsville, AL). Viral primers originating from [12,14] were used to amplify fragments of the respective viral polymerases (Table 1). [Note: SCN negative-sense virus reverse (R) primers are oriented to the viral genome and reverse universal (RU) primers are oriented to the mRNA]. HgFAR1 was used as an internal control for SCN as this gene was used by [14] for qRT-PCR. Additionally, a primer pair for GAPDH (NCBI Genbank accession number CA939315) was designed as this gene is validated for steady expression throughout different SCN life stages by [19]. When testing soybean plants for virus, the *Glycine max* internal control gene ELF1B was amplified via primers from [20] listed in Table 1. qRT-PCR products were amplified using 0.5 μM of each appropriate primer pair, 10 μl iTaq^™^ Universal SYBR^®^ Green Supermix (Bio-Rad, Hercules, CA), and 1 μl cDNA. Applied Biosystems 7000 Real-Time PCR System was used at the following settings: 95°C, 10 minutes; 95°C, 15 seconds; 60°C, 1 minute for a total of 40 amplification cycles with a melt curve of 60°C for 20 seconds and 95°C for 15 seconds. Plant products were amplified utilizing the same methodology but with the newer technology of the Applied Biosystems QuantStudio™ 6 Flex Real-Time PCR system on the following settings: 95°C, 20 seconds; 95°C, 2 seconds; 60°C, 25 seconds repeated for 40 amplification cycles with a continuous melt curve of 95°C, 20 seconds; 60°C, 1 minute; and 94°C for 20 seconds.

Cycle threshold values (Ct; amplification cycle in which fluorescence emitted exceeds background fluorescence) equal to or greater than 35 were considered non-detectable. DNase treatments yielded insignificant results between Ct values of treated and untreated samples and was not necessary for analysis. The average normalized abundance ratios (i.e. relative amount of virus in each SCN sample) were determined for each population sample. Ct values of SCN viruses were normalized against the mean Ct values of SCN internal reference genes (GAPDH and/or HgFAR1) using the equation E_internal_^Ct(internal)^/E_target_^Ct(target)^ where E equals the efficiency of a primer pair [21]. Further modifications were made for addressing viral titer compared with host internal control genes [22]. Primer efficiencies were calculated by the equation 2^(-1/slope)^ via a five-point 1:2 dilution series. The efficiencies of primer pairs are E_ScNV_ = 1.91 (91%), E_ScPV_ = 1.92 (92%), E_ScRV_ = 1.88 (88%), E_ScTV_ = 1.89 (89%), E_SbCNV-5_ = 1.98 (98%), E_HgFAR1_ = 2.05 (105%), and E_GAPDH_ = 2.07 (107%). Average normalized abundance of field populations were calculated using HgFAR1 as the internal control due to limited sample material; greenhouse populations were analyzed with both HgFAR1 and GAPDH. The average difference between GAPDH and HgFAR1 mean Ct values for greenhouse populations of SCN eggs was 0.83, less than one amplification cycle. Field samples for which both internal controls were amplified had Ct values that differed by a mean of 0.37. Further statistical analysis of samples was conducted via GraphPad Prism 6 software (GraphPad Software, La Jolla, CA).

### Viral presence and replication in different SCN life stages

OP50 and PA3 populations of SCN were analyzed for viral titer and replication within different life stages; these SCN lines were maintained at an NCSU greenhouse on susceptible soybean hosts. For each biological replication, four plants grown in individual cone planters were pooled for nematode extraction one month after inoculation with SCN eggs. Eggs were extracted from SCN females and collected via stacked sieves and sucrose centrifugation as previously described. Approximately half of the collected eggs were reserved for RNA extraction. The remaining eggs were hatched for three days at 27°C using a Baermann pan method [23] and the cohort of hatched J2 were collected. The remaining infected soybean roots were pulverized in a blender to remove J3 and J4 stages of SCN and the nematode sample was cleaned via stacked sieves and sucrose centrifugation. Pooled J3 and J4 samples of SCN were further concentrated by pelleting the collected nematodes in a microcentrifuge tube. RNA extraction from the collected SCN eggs, hatched J2, and J3/J4 life stages was completed as described above.

First strand cDNA synthesis was initiated with either random, genomic sense, or anti-genomic sense primers. Genomic sense primers used to initiate cDNA were ‘F500’ series and anti-genomic sense primers were ‘RU’ series. ‘Q’ primer pairs were utilized for qRT-PCR analysis as these primers are nested between the ‘F500’ and ‘RU’ primers (Table 1). OP50 samples were analyzed on an Applied Biosystems 7000 Real-Time PCR System, while PA3 samples were analyzed on an Applied Biosystems QuantStudio™ 6 Flex Real-Time PCR System under the previously described settings. qRT-PCR average relative abundance ratios were quantified using the SCN internal control GAPDH as previously described via the Pfaffl method [21]. Pooled technical triplicates were electrophoresed on a 2% TAE agarose gel for further analysis.

### Virus detection in other PPN species via qRT-PCR

New viral primers were designed to account for a potentially high degree of sequence dissimilarity for viruses in other PPN species. Therefore, viral ‘Q’ primers were designed within conserved regions of 500 nucleotide sequences of viral polymerases (Table 1). The nematode species *Heterodera schachtii* (beet cyst), *H*. *trifolii* (clover cyst), *Vittatidera zeaphila* (corn cyst) [24], *Globodera rostochiensis* (golden potato cyst), *G*. *tabacum* (tobacco cyst) were tested for viruses. Additionally, *Meloidogyne arenaria* (peanut root-knot), *M*. *hapla* (northern root-knot), *M*. *incognita* (southern root-knot), *M*. *javanica* (Javanese root-knot), and *Rotylenchus reniformis* (reniform) were examined. Due to either unavailable or dissimilar GAPDH sequences across nematode species, internal control primers were designed from conserved regions of the 18*S* ribosomal RNA gene among sequences available from NCBI Genbank including *H*. *glycines* (AY043247), *H*. *schachtii* (KJ636284), *Meloidogyne javanica* (AF442193) and *Aphelenchoides besseyi* (AY508035). The resulting primers are also listed in Table 1. Products were analyzed on an Applied Biosystems QuantStudio™ 6 Flex Real-Time PCR System under the previously described settings. Samples positive for viruses were electrophoresed on a 2% TAE agarose gel.

### Confirmation of viral presence via endpoint PCR and Sanger sequencing

To confirm positive qRT-PCR results, a 500 bp region of the appropriate viral polymerases were amplified. The reverse universal (RU) primers from [12] and SbCNV-5-R primer [14] were used in conjunction with newly designed ‘F500’ primers (Table 1) to amplify an approximately 500 bp PCR product. OneTaq^®^ 2X Master Mix with standard buffer (New England BioLabs) and appropriate primers were used according to manufacturer’s protocol to amplify products from cDNA in the Bio-Rad C1000 Touch Thermal Cycler under the following conditions: 94°C, 5 minutes; 94°C, 30 seconds; 60°C, 30 seconds; 68°C, 40 seconds for 40 amplification cycles followed by a final extension of 68°C for 5 minutes. Products were electrophoresed on a 1% TAE agarose gel with 1X TAE buffer. PCR products were purified for sequencing with the QIAquick® PCR Purification Kit (Qiagen, Valencia, CA) according to manufacturer’s instructions. In the case of low viral titers, PCR products were cloned into the pGEM®-T Easy Vector System (Promega, Madison, WI) and purified from transformed *Escherichia coli* via QIAprep® Spin Miniprep Kit (Qiagen). Sanger sequencing was performed by Eurofins Genomics with sequence primers listed on S1 Table. Sequence results were analyzed using Vector NTI Advance^®^ 11.5.3 software (Invitrogen).

## Results

### Viruses are not detectable in soybean plants

The SCN viruses are most closely related to viruses that are transmitted from insects to plants, but these specific viruses have not been reported to infect plants. Because of this potential, the pods, leaves, stems, and roots of SCN infected and noninfected soybean plants were collected for analysis via qRT-PCR. The soybean cultivars Bedford, Hartwig, Jake, NC Raleigh, NC Roy and Williams 82 were tested for viruses via qRT-PCR (roots were tested only for soybean noninfected with SCN). Furthermore, split root trials with the soybean variety NC Roy were conducted where one-half of the root system was exposed to SCN. Roots were infected with either LY1 (harbors five known SCN viruses) or MM21 (noninfected with viruses), and roots from the noninfected half of the root system were tested for SCN viruses. The internal control gene ELF1B (eukaryotic elongation factor I-beta) [20] was used as a measure of soybean genetic material via qRT-PCR. Additionally, SCN primers (GAPDH and 18*S*) were utilized to further rule out nematode contamination; all attempts to amplify GAPDH and 18*S* were unsuccessful. SCN viruses were not detectable in any quantity within soybean plant tissues suggesting that these viruses are not passed from nematode to soybean plant or vice versa. If roots are capable of being infected by SCN viruses, infection would most likely be localized and not move throughout the root system or plant. Future testing of the root closer to a nematode feeding site may determine if a small site of localized infection exists.

### Viruses are present and actively replicating in different SCN life stages

**Fig 1:**
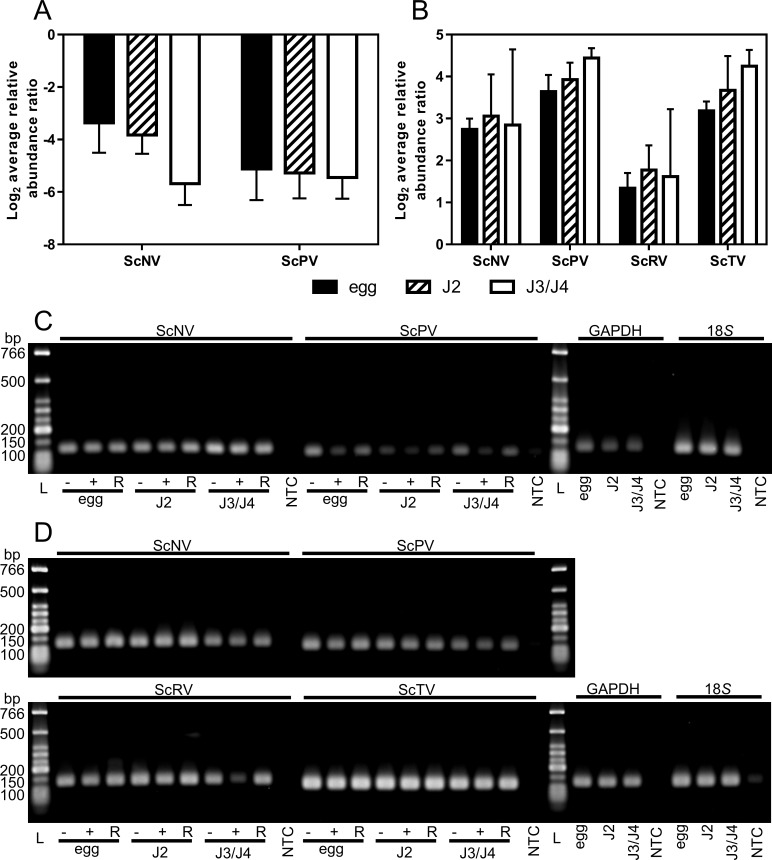
Titer and replication of negative-sense RNA viruses within SCN life stages measured with qRT-PCR. Log_2_ average relative abundance of viruses within SCN OP50 (**A**) and PA3 (**B**) populations for egg, J2 and J3/J4 stages. Each experiment was conducted in technical triplicates and two different biological replicates. Error bars represent the SEM. Analysis of the means was conducted with 2-way ANOVA (GraphPad Prism 6). Active replication of viruses is shown for OP50 (**C**) and PA3 (**D**) by analyzing qRT-PCR products on a 2% agarose gel. The following abbreviations are explained: low molecular weight ladder (L; New England BioLabs), no template control (NTC), genomic RNA (-), anti-genomic RNA (+) and both genomic and anti-genomic RNA control initiated with random primers. Internal controls for SCN are GAPDH and 18*S*.

The egg, J2, and pooled J3/J4 stages of SCN were examined via qRT-PCR to see if viruses are detectable within multiple nematode life stages. Two different greenhouse lines of SCN (OP50 and PA3) were used in this experiment as these populations are commonly used for research purposes. OP50 (population originating from an NCSU greenhouse) was found to be infected with ScNV and ScPV via qRT-PCR (Fig 1A) whereas, PA3 harbors viral infections of ScNV, ScPV, ScRV and ScTV (Fig 1B). Viruses were detected in all SCN life stages tested and there were no significant differences in viral titers when comparing across multiple life stages of OP50 or PA3. Within a particular life stage, there was also no single virus that was expressed at a significantly higher or lower titer than another virus. These data suggest that the viruses detected are stably present throughout the life stages of SCN. The Ct values and average normalized abundance ratios are reported for OP50 (S2 Table) and PA3 (S3 Table).

To deduce whether viruses (ScNV, ScPV, ScRV and ScTV) actively replicate within the nematode, first strand cDNA synthesis was initiated with primers specific to either the genomic or anti-genomic sense strand of RNA. qRT-PCR products internal to the cDNA primer initiation sites were amplified (S4 Table), and an electrophoresis gel of viral PCR products from OP50 demonstrated that both RNA-sense strands are amplified for ScNV and ScPV (Fig 1C). ScNV genomic and anti-genomic products are visible at relatively similar concentrations while anti-genomic RNA of ScPV is present at a visibly lower concentration in OP50. Typically, non-symmetric replication of the viral genome versus the anti-genome is expected. In the case of ScNV, viral titer may be high enough that differences in abundance cannot be visualized via electrophoresis of qRT-PCR products. Normalized abundance ratios were not calculated for this experiment as different cDNA was used to amplify specific orientations of viral products and internal controls were only amplified from cDNA initiated with random primers. The experiment was also replicated with PA3 (Fig 1D; S5 Table) and similar results are observable. For the four viruses present in PA3, both genomic and anti-genomic sense RNAs are detectable in egg, J2, and J3/J4 life stages. Anti-genomic RNA is visibly lower for ScRV within the PA3 J3/J4 life stages as compared with genomic RNA. ScNV, ScPV and ScTV titers are high for both negative and positive-sense RNAs in PA3. The presence of anti-genomic RNAs demonstrates that negative-sense RNA viruses are actively replicating within multiple life stages of SCN.

### Viruses are prevalent in NC and MO greenhouse cultures and field populations of SCN

**Fig 2:**
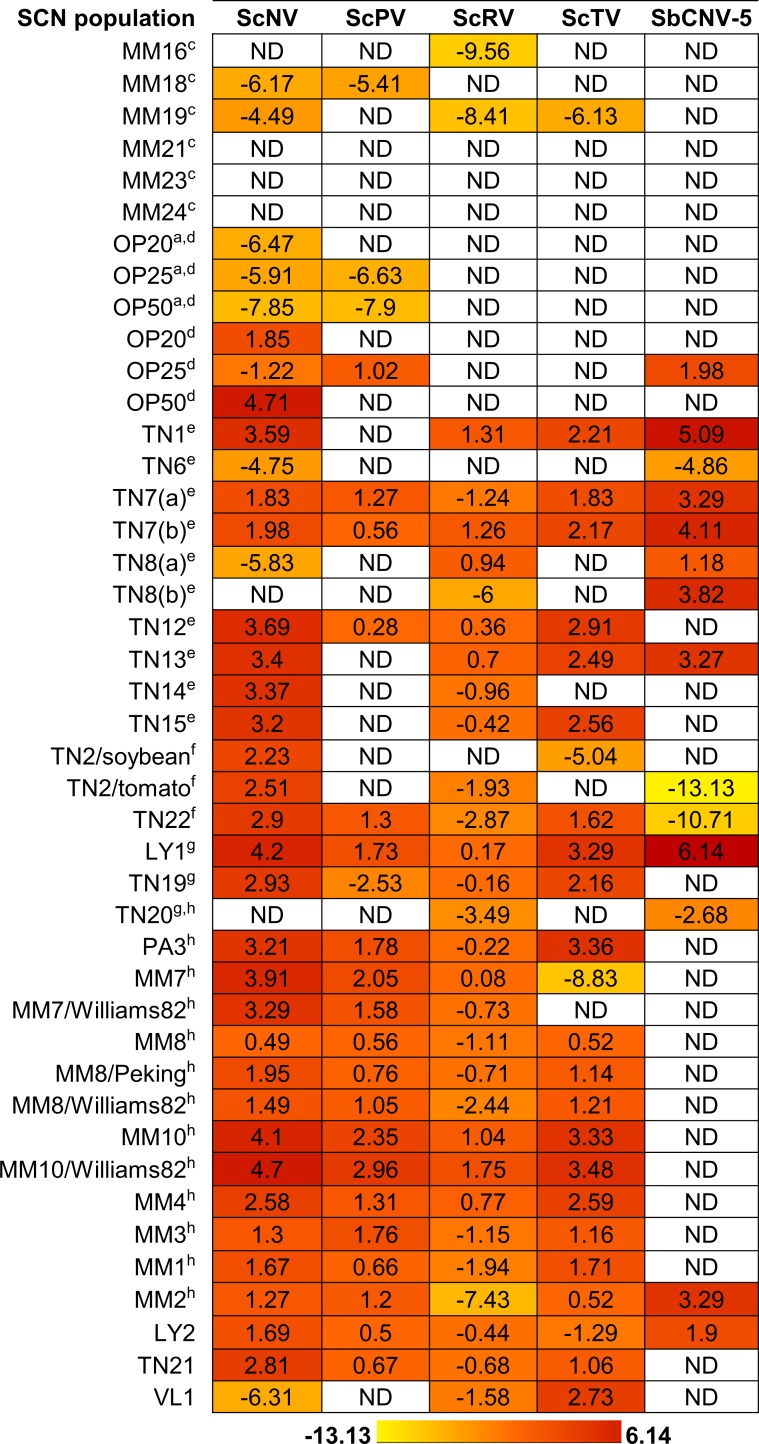
Log_2_ average relative abundance ratios of respective viruses in SCN egg samples from populations maintained in research greenhouses. Viral presence and level were determined by relative quantification of qRT-PCR values normalized against SCN internal control genes HgFAR1 and GAPDH. Experiments were conducted in technical triplicates. The color gradient represents a heat map of variance in viral titers.

Forty-seven SCN greenhouse populations were surveyed for viruses to expand upon previous work in which viruses were detected in a limited number of SCN inbred greenhouse lines [12,14]. A small subset of the SCN lines—OP20, OP25, OP50 [25]—had been inbred and maintained in NCSU greenhouses for almost twenty years. The remaining greenhouse SCN populations tested were maintained within greenhouses at the University of Missouri (MU) and had originated from different field sites and inbred over time (Table 2). Total RNAs were extracted from SCN eggs followed by first strand cDNA synthesis and qRT-PCR analysis. Fig 2 displays the SCN greenhouse populations surveyed and heat map of the approximate viral titer (i.e. average normalized abundance ratio) within nematode egg samples relative to internal nematode control genes HgFAR1 and GAPDH. Average Ct values for greenhouse populations are listed in S6 Table. Each of the five SCN viruses were found frequently in inbred SCN greenhouse populations. Five SCN populations (TN7, TN22, LY1, MM2 and LY2) harbored infections with all five viruses known for SCN. Contrastingly, several SCN populations (MM21, MM23 and MM24) of similar descent (Table 2) have no detectable viral infections. A mixture of viral infections was present in the majority of SCN populations that were tested. There does not appear to be a distinct pattern of viral infections correlated with nematode virulence. For example, PA3 is SCN Hg type 0 and MM10, which possesses similar viral titers, is Hg type 1.2.3.4.5.6.7. ScRV and ScTV were only detected in SCN cultures originating in MO, and SbCNV-5 was also detected in greater frequency in SCN cultures from MO compared with SCN cultures originating from NC.

**Fig 3:**
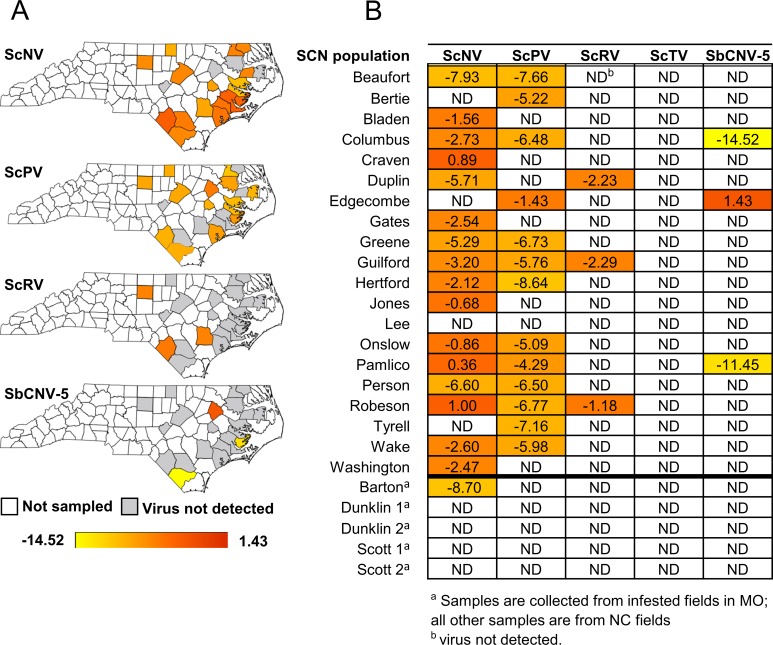
Log_2_ average relative abundance ratios of respective viruses in SCN egg samples from NC and MO infested fields. Viral titers are represented spatially (**A**) and numerically for NC county field samples (**B**). Virus presence and level are determined by relative quantification of qRT-PCR values normalized to SCN internal control gene HgFAR1. Experiments were conducted in technical triplicates. The color gradient accentuates differences in viral titers.

In addition to greenhouse populations, 25 field populations of SCN in NC and MO were sampled to deduce whether viral infections exist and are prevalent in an agricultural setting. Due to the geography and climate of NC, soybeans are grown profitably almost exclusively in the eastern third of the state. The distribution of NC counties sampled for SCN viruses are mapped in Fig 3A. Twenty field samples, each from twenty different NC counties, yielded enough total RNA to reliably detect SCN internal control genes. There does not appear to be any clear pattern of viral distribution across NC; however, there does appear to be natural viral infections of SCN in agricultural fields. Approximate viral titers of county samples measured by qRT-PCR are listed and heat mapped in Fig 3. ScNV was the most common and highest titer virus found in SCN from NC fields followed by ScPV. The majority of viral titers in NC field samples of SCN were considered as non-detectable (ND), falling below the expression level of the reference SCN gene (GAPDH). Additionally, five field samples of SCN from MO were sampled to deduce whether viruses were present within another geographic area and if frequencies of viral infections differed. In the MO field samples of SCN tested, ScNV was the only virus detectable within one of five populations and is lower titer than the majority of NC samples. qRT-PCR Ct values for field samples are provided in S7 Table.

**Fig 4:**
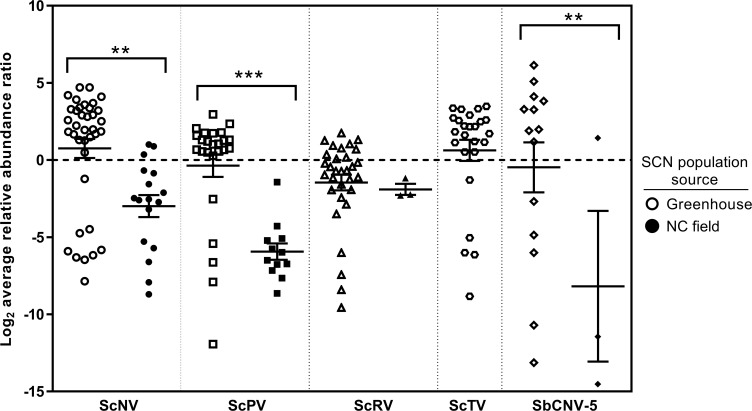
Log_2_ average relative abundance ratios of SCN samples in which virus is detectable via qRT-PCR. Both SCN greenhouse and NC field population values are shown except for ScTV as this virus was not detected in field samples. The center line denotes the mean and error bars represent the SEM. Analysis of the means was conducted with 2-way ANOVA (GraphPad Prism 6) and asterisks demonstrate significant differences at p = .01 (**) and p = .001 (***) levels.

The percentage of SCN samples infected with ScNV or ScPV is similar for both greenhouse and field populations (Table 3). The biggest discrepancy in viral presence between these population sources is that ScTV is detectable within a high percentage of greenhouse populations but is not found within any NC or MO field samples. ScRV and SbCNV-5 are less frequently detected within field samples of SCN. These results suggest these viruses, with the exception of perhaps ScTV, are not artifacts of inbreeding greenhouse populations of SCN. Moreover, there are no significant differences between mean SCN viral titers of field and greenhouse populations of ScRV (Fig 4). Conversely, mean viral titer levels are significantly higher for ScNV, ScPV and SbCNV-5 in SCN greenhouse populations. Thus, the replication process of ScRV does not appear to be hindered within field isolates of SCN. ScNV, ScPV and SbCNV-5 titers were lower in SCN field populations. However, for SbCNV-5 this could be a statistical result from relatively few samples being infected by this virus. Notably, ScTV was not detected within field samples from NC or greenhouse populations of NC origin [OP20, OP25 and OP50] even when maintained in MU greenhouses. Future work will examine if NC populations of SCN have innate immunity to ScTV or if these populations simply have not yet been exposed to this virus.

**Table 3:** Number and percent of North Carolina SCN greenhouse and NC field population samples infected with viruses as detected with qRT-PCR.

	Greenhouse populations		NC field populations	
Virus	Number infected	Percent infected	Number infected	Percent infected
ScNV	38/43	88.4%	16/20	80.0%
ScPV	25/43	58.1%	13/20	65.0%
ScRV	31/43	72.1%	3/20	15.0%
ScTV	25/43	58.1%	0/20	0.0%
SbCNV-5	14/43	32.6%	3/20	15.0%

### Other cyst nematode species are infected with SCN viruses

**Fig 5:**
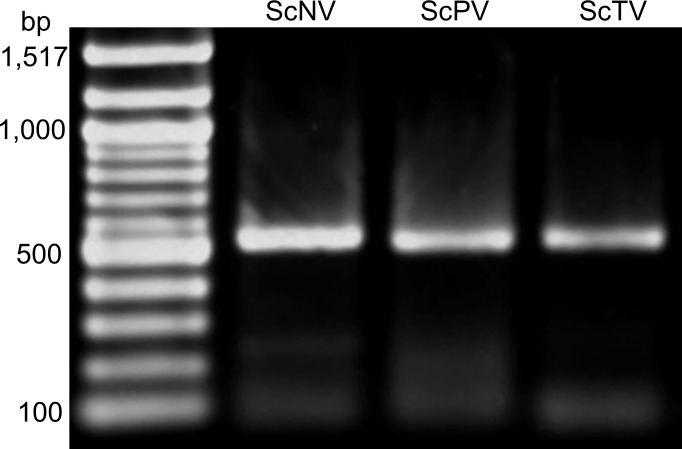
Amplification of SCN viruses within *Heterodera trifolii*. PCR products of the approximately 500 bp RdRp region for ScNV, ScPV and ScTV were amplified from total RNA extracted from *H*. *trifolii* and electrophoresed on a 1% agarose gel with 1 kb molecular ladder (New England BioLabs).

Other PPN species were sampled for the presence of the known five viruses to see if viral host ranges extended beyond SCN. Each nematode sample was tested for viruses via qRT-PCR using the “Q” primer sets (Table 1) and conditions that were used for SCN. No viruses were detectable in the majority of tested PPN species. Only clover cyst nematode (*H*. *trifolii*) and beet cyst nematode (*H*. *schachtii*) harbor three or two SCN viruses, respectively (Table 4) as detected by the PCR assay used here. Additional cyst (*Globodera rostochiensis*, *G*. *tabacum*, *Vittatidera zeaphila*), root-knot (*Meloidogyne arenaria*, *M*. *hapla*, *M*. *incognita*, *M*. *javanica*), and reniform (*Rotylenchus reniformis*) nematode species did not possess SCN viruses at detectable levels. Normalized abundance ratios were not calculated for this experiment as a new internal control (18*S*) was utilized due to sequence dissimilarity among PPN species. Positive qRT-PCR results were then confirmed by endpoint PCR amplification and Sanger sequencing of a 500 bp region of the RNA-dependent RNA polymerase (RdRp). *H*. *trifolii* was confirmed to possess ScNV, ScPV and ScTV (Fig 5) sequences. In two separate *H*. *schachtii* samples, a faint band for the 500 bp product was seen for either ScPV or ScRV (images not shown). The PCR products of *H*. *schachtii* were also confirmed to be the correct virus products by Sanger sequencing.

**Table 4:** Detection of known SCN viruses in other species of plant-parasitic nematodes via qRT-PCR.

				Average Ct value^a^					
PPN species	Life stage	Location	Date collected	ScNV	ScPV	ScRV	ScTV	SbCNV -5	18*S*^b^
*Globodera rostochiensis*	egg	CU	2015	ND^c^	ND	ND	ND	ND	13.22
	J2	CU	2015	ND	ND	ND	ND	ND	13.30
*G*. *tabacum*	egg	MU	2015	ND	ND	ND	ND	ND	11.51
	J2	NCSU	1997a	ND	ND	ND	ND	ND	11.17
			1997b	ND	ND	ND	ND	ND	9.435
*Heterodera schachtii*	J2	NCSU	2008	ND	30.75	ND	ND	ND	12.90
			2009a	ND	ND	ND	ND	ND	15.65
			2009b	ND	ND	ND	ND	ND	12.88
			NA^d^	ND	ND	34.60	ND	ND	11.60
*H*. *trifolii*	egg	MU	2014	25.81	23.25	ND	26.97	ND	13.45
*Meloidogyne arenaria*	J2	NCSU	1997	ND	ND	ND	ND	ND	14.16
			NA	ND	ND	ND	ND	ND	9.489
*M*. *hapla*	J2	NCSU	1997	ND	ND	ND	ND	ND	7.688
*M*. *incognita*	J2	NCSU	1994a	ND	ND	ND	ND	ND	15.46
			1994b	ND	ND	ND	ND	ND	6.926
			2001	ND	ND	ND	ND	ND	12.70
			NAa	ND	ND	ND	ND	ND	9.114
			NAb	ND	ND	ND	ND	ND	8.072
*M*. *javanica*	J2	NCSU	1995	ND	ND	ND	ND	ND	7.403
*Rotylenchus reniformis*	J2	NC field	2003	ND	ND	ND	ND	ND	13.79
			NA	ND	ND	ND	ND	ND	12.49
*Vittatidera zeaphila*	egg	MU	2015	ND	ND	ND	ND	ND	10.93

^a^ average of technical triplicates.

^b^ internal control.

^c^ not detectable.

^d ^date not available.

## Discussion

Until recently, nematodes were widely considered noninfected by viruses primarily due to a scarcity of convincing published reports. High-throughput genomic and transcriptomic sequencing has enabled discovery of naturally occurring viruses within *C*. *elegans* and *C*. *briggsae* [9] and SCN [12,14]. This study confirms via qRT-PCR and Sanger sequencing of viral RdRp segments, the reports [12,14] that five viruses are present within SCN from different geographic regions. The results demonstrated that viruses are present within greenhouse populations of SCN, including extensive analysis of greenhouse populations commonly used in research. We report the presence of negative-sense viruses for the first time in field populations of SCN from two different states, NC and MO. Moreover, the negative-sense viruses are shown to actively replicate within the SCN host throughout egg, J2, and J3/J4 life stages. Finally, viruses are reported for the first time within the closely related cyst nematode species *H*. *trifolii* and *H*. *schachtii*.

Viruses are present within both greenhouse and field populations of SCN. Infection rates of ScNV and ScPV were similar for greenhouse and NC field samples (Table 3). The starkest difference in viral presence is ScTV that was detected in 58.1% of greenhouse populations but is not found in field samples. Future studies could examine whether NC SCN can be infected if introduced to ScTV or if the tested field populations are immune to infection by this particular virus. Interestingly, for those samples that tested positive, viral titers were not significantly different between field and greenhouse samples for ScRV (Fig 4). However, titers were higher for ScNV, ScPV and SbCNV-5 in greenhouse populations. SbCNV-5 values do not cluster which may be a result of a small sample size or variation within SbCNV-5 replication itself. These data are similar to the findings of [14] who found SbCNV-5 in greenhouse populations but detected the virus less commonly in the field. Previously [12], field samples were not analyzed for the four negative-sense RNA viruses but did detect these viruses in several greenhouse lines of SCN. Preliminary results suggest SCN viruses could be more common in NC than in MO field samples as ScNV is the only virus detected in one of the five MO field samples. One possible explanation for this observation is that NC field samples were introduced to viruses when population levels were amplified in NCSU greenhouses for several months. However, this scenario is unlikely as several NC field samples tested positive for ScNV and ScPV when sampled directly from the field. A larger sampling of fields in MO and other geographic areas may illustrate a clearer picture of SCN viral infections. Moreover, viral titers are lower for SCN populations maintained in NCSU greenhouses as compared with those at MU. OP20, OP25 and OP50 are maintained at both locations, but virus levels are higher in MU populations. Greater inbreeding of greenhouse populations (i.e. TN, MM lines) among the MU SCN lines seemed to increase both virus frequency and titer, while the opposite seemed true for the inbred (OP) lines at NCSU. These discrepancies raise questions about how SCN viruses are transmitted and if different greenhouse conditions and research techniques can alter viral composition. Further confounding the issue, viral titers may vary within a particular population because of time of sample collection, soil temperature or host plant.

Related groups of nematodes seem to harbor similar infections. For instance, TN20 and PA3 are the parent lines of MM7, MM8 and MM10 (Fig 2) [26]. Subsequent populations are similar to PA3 in viral content and possess high titers of the negative-sense RNA viruses. Similarly, the related populations MM16, MM18, MM19, MM21, MM23 and MM24 represent a relatively recent inbred MO field population and these nematodes have low amounts or no virus present. Although the titers are lower, the types of viral infections are different for MM16, MM18 and MM19. This inconsistency may arise because additional viruses are actually present but are below the detection limits of qRT-PCR. Another possibility is that viral titers are similar because these particular nematode populations have a genetic disposition to limit viral replication. Much of the available literature examines viruses of non-pathogenic *C*. *elegans* and *C*. *briggsae* [9,10] and makes it difficult to determine if viruses could affect nematode virulence. However, from this study it does not appear that viral infection is related to SCN virulence. As noted earlier, SCN lines with similar types and amounts of viral infection can have dissimilar HG types. One of the least virulent lines, PA3, has an HG type of 0 and one of the most virulent lines, MM10, has an Hg type of 1.2.3.4.5.6.7; yet, PA3 and MM10 are infected with ScNV, ScPV, ScRV and ScTV at similar titers.

Negative-sense RNA viruses are present within egg, J2, and J3/J4 stages of SCN. There are no significant differences in viral titers between life stages (Fig 1A and 1B). Furthermore, RNA viruses actively replicate within immature life stages of SCN (Fig 1C and 1D). Anti-genomic sense RNAs are detectable for ScNV, ScPV, ScRV and ScTV denoting that the viral genomes are being replicated. These data agree and expand upon the work of [12] who used a positive strand specific qRT-PCR assay to suggest that SCN negative-sense viruses replicate in the host. Although viral production did not increase within immature stages, future studies will examine if viral titers are higher within males or virgin females just prior to mating. Additionally, if the virus is nonpathogenic to SCN, then a stable, low titer of virus may be tolerated by the nematode innate immune system.

Since variation was observed among the 500 bp sequences of viral polymerases, new qRT-PCR primers were designed within conserved regions of the known SCN viruses to increase potential detection of these viruses within other PPN species. Additional PPN species were tested for viruses (Table 4) with only cyst nematode species *H*. *schachtii* and *H*. *trifolii* harboring detectable viruses. An approximately 500 bp region of each viral RdRp was confirmed to be the correct PCR product via Sanger sequencing. Three viruses were found within *H*. *trifolii*: ScNV, ScPV and ScTV. The viruses ScPV and ScRV are present in two separate *H*. *schachtii* population samples from 2008 and an unknown date (Table 4). It is not unrealistic to believe SCN viruses are present within *H*. *schachtii* and *H*. *trifolii*. The phylogenic relationship of the *Schachtii* lineage is reported [27] based upon ITS regions of ribosomal DNA sequences. Coincidentally, *H*. *glycines* is most closely related to *H*. *trifolii* followed by *H*. *mediterranea* (not tested) and *H*. *schachtii*. These data suggest SCN viruses may have evolved within this subset of cyst nematodes before the species split from one another. Additional analysis of viral genomes in PPN may resolve relationships between different virus isolates. Furthermore, it is possible that viruses discovered in SCN exist in additional nematode species but the titers are below detectable limits, primers cannot bind due to genetic dissimilarity or they simply have not yet been tested. More objective analyses to detect potential virus infection of other PPN species are certainly warranted given the extent of virus infection that occurs in SCN.

Predicting why there is variance in viral infections among populations and environments without knowing the mode of transmission or tropisms is challenging. There may be a dilution effect if the viruses are localized to only a few cells in comparison to the internal control (GAPDH) that expresses a single copy in each cell. The information available on *Caenorhabditis* viruses may help to localize SCN viral infections within the nematode. Viruses that infect *Caenorhabditis* species were discovered to possess tropism to several intestinal cells [11]. Methods of natural transmission have not been clearly deduced; however, experimental infection of *C*. *elegans* with FHV is possible by microinjection of *C*. *elegans* and FHV plasmids into the gonad [8]. Unfortunately, this technique does not explain how viruses naturally establish infection in nematodes.

Future work will determine if nematode viruses are contributing factors to host development or pathogenicity. Relatedly, treating human infections of the nematode species *Onchocerca volvulus* with doxycycline in conjunction with ivermectin enables greater treatment success. The antibiotic sterilizes female nematodes by attacking an endosymbiotic bacterium, *Wolbachia spp*., which controls host development [28]. If viruses can function similarly in other parasitic nematodes, targeting viruses could help to disrupt the parasitic cycles of important nematode species. More in depth studies of microbial symbionts of nematodes may yield interesting results that can further development of novel control strategies, and it is likely that future next-generation sequencing projects will continue to unveil additional novel viral sequences within nematode species. Although SCN viruses were not detectable in soybean plant tissue, more intensive testing of SCN weed hosts may yield interesting results as to additional hosts for these viruses. From the presented research, we hope to demonstrate that nematode viruses are more widespread than previously thought and may have important impacts on nematode biology. Thus in the future, viruses could prove useful for development of novel biological controls to target nematodes. Upcoming studies include examining how viral transmission occurs and localizing cellular and tissue tropisms.

## Supporting Information

S1 Table Sequence primers of approximately 500 bp regions of viral RdRPs via Sanger sequencing. (DOCX)

S2 Table Average Ct values for detectable viruses in SCN (OP50) life stages egg, J2, and mixed J3/J4 stages. The Ct value of each virus was normalized against the SCN internal control gene GAPDH.
(DOCX)

S3 Table Average Ct values for detectable viruses in SCN (PA3) life stages egg, J2, and J3/J4 stages. The Ct value of each virus was normalized against the SCN internal control gene GAPDH.
(DOCX)

S4 Table Mean qRT-PCR Ct value for SCN (OP50) egg, J2 and J3/J4 stages used to determine viral replication in SCN. Data presented are the means of technical triplicates. Genomic and anti-genomic RNA was detected by initiating first strand cDNA synthesis with primers specific to each strand. Random primers were also used for cDNA synthesis as a control for both genomic and anti-genomic RNA.
(DOCX)

S5 Table Mean qRT-PCR Ct value for SCN (PA3) egg, J2, and J3/J4 stages used to determine viral replication in SCN. Data presented are the means of technical triplicates. Genomic and anti-genomic RNA was detected by initiating first strand cDNA synthesis with primers specific to each strand. Random primers were also used for cDNA synthesis as a control for both genomic and anti-genomic RNA.
(DOCX)

S6 Table Mean of qRT-PCR Ct values of SCN greenhouse populations. Data presented are the means of technical triplicates. SCN internal controls for relative quantification are HgFAR1 and GAPDH.
(DOCX)

S7 Table Mean of qRT-PCR Ct values of field populations of SCN. Data presented are the means of technical triplicates. The SCN internal control HgFAR1 is used for relative quantification of virus abundance.
(DOCX)

## References

[1] Brown DJ, Robertson WM, Trudgill DL. Transmission of viruses by plant nematodes. Annu Rev Phytopathol. 1995; 33: 223–249. 10.1146/annurev.py.33.090195.001255 18999960

[2] Gray SM, Banerjee N. Mechanisms of arthropod transmission of plant and animal viruses. Microbiol Mol Biol Rev. 1999; 63: 128–148. 10066833 10.1128/mmbr.63.1.128-148.1999 PMC98959

[3] Hooper DJ. Virus vector nematodes–taxonomy and general introduction In: Lamberti F, Taylor CE, Seinhorst JW, editors. Nematode vectors of plant viruses. London: Plenum Press; 1974 pp 1–14.

[4] Loewenberg JR, Sullivan T, Schuster ML. A virus disease of *Meloidogyne incognita incognita*, the southern root knot nematode. Nature. 1959; 184: 1896 14417916 10.1038/1841896a0

[5] Foor WE. Virus-like particles in a nematode. J Parasitol. 1972; 1065–1070. 4641873

[6] Poinar GO, Hess R. Virus-like particles in the nematode *Romanomermis culicivorax* (Mermithidae). Nature. 1977; 266: 256–257. 846569 10.1038/266256a0

[7] Zuckerman BM, Himmelhoch S, Kisiel M. Virus-like particles in *Dolichodorus heterocephalus*. Nematologica. 1973; 19: 117.

[8] Lu R, Maduro M, Li HW, Broitman-Maduro G, Li WX, Ding SW. Animal virus replication and RNAi-mediated antiviral silencing in *Caenorhabditis elegans*. Nature. 2005; 436: 1040–1043. 10.1038/nature03870 16107851 PMC1388260

[9] Felix MA, Ashe A, Piffaretti J, Wu G, Nuez I, Belicard T, et al Natural and experimental infection of *Caenorhabditis* nematodes by novel viruses related to nodaviruses. PLoS Biol, 2011; 9: e1000586 10.1371/journal.pbio.1000586 21283608 PMC3026760

[10] Franz CJ, Zhao G, Félix MA, Wang D. Complete genome sequence of Le Blanc virus, a third *Caenorhabditis* nematode-infecting virus. J Virol. 2012; 86: 11940–11940. 10.1128/JVI.02025-12 23043172 PMC3486331

[11] Franz CJ, Renshaw H, Frezal L, Jiang Y, Felix MA, Wang D. Orsay, Santeuil and Le Blanc viruses primarily infect intestinal cells in *Caenorhabditis* nematodes. Virology. 2013; 448: 255–264. 10.1016/j.virol.2013.09.024 24314656

[12] Bekal S, Domier LL, Niblack TL, Lambert KN. Discovery and initial analysis of novel viral genomes in the soybean cyst nematode. J Gen Virol. 2011; 92: 1870–1879. 10.1099/vir.0.030585-0 21490246

[13] Wrather JA, Koenning SR. Estimates of disease effects on soybean yields in the United States 2003 to 2005. J Nematol. 2006; 38: 173–180. 19259444 PMC2586459

[14] Bekal S, Domier LL, Gonfa B, McCoppin NK, Lambert KN, Bhalerao K. A novel flavivirus in the soybean cyst nematode. J Gen Virol. 2014; 95: 1272–1280. 10.1099/vir.0.060889-0 24643877

[15] Colgrove AL, Niblack TL. Correlation of female indices from virulence assays on inbred lines and field populations of *Heterodera glycines*. J Nematol. 2008; 40: 39–45. 19259518 PMC2586527

[16] Koenning SR, Barker KR. Survey of *Heterodera glycines* races and other plant-parasitic nematodes on soybean in North Carolina. J Nematol. 1998; 30: 569–576. 19274248 PMC2620330

[17] Mitchum MG, Wrather JA, Heinz RD, Shannon JG, Danekas G. Variability in distribution and virulence phenotypes of *Heterodera glycines* in Missouri during 2005. Plant Dis. 2007; 91: 1473–1476. 10.1094/PDIS-91-11-1473 30780744

[18] Chomczynski P, Sacchi N. Single step method of RNA isolation by acid guanidinium thiocyanate-phenol-chloroform extraction. Anal Biochem. 1987; 162: 156–159. 10.1006/abio.1987.9999 2440339

[19] Ithal N, Recknor J, Nettleton D, Hearne L, Maier T, Baum TJ, et al Parallel genome-wide expression profiling of host and pathogen during soybean cyst nematode infection of soybean. Mol Plant-Microbe Interact. 2007; 20: 293–305. 10.1094/MPMI-20-3-0293 17378432

[20] Jian B, Liu B, Bi Y, Hou W, Wu C, Han T. Validation of internal control for gene expression study in soybean by quantitative real-time PCR. BMC Mol Biol. 2008; 9: 59 10.1186/1471-2199-9-59 18573215 PMC2443375

[21] Pfaffl MW. A new mathematical model for relative quantification in real-time RT–PCR. Nucleic Acids Res. 2001; 29: 2002–2007. 10.1093/nar/29.9.e45 PMC55695 11328886

[22] Rotenberg D, Krishna Kumar NK, Ullman DE, Montero-Astúa M, Willis DK, German TL, et al Variation in Tomato spotted wilt virus titer in *Frankliniella occidentalis* and its association with frequency of transmission. Phytopathology. 2009; 99: 404–410. 10.1094/PHYTO-99-4-0404 19271982

[23] Townshend JL. A modification and evaluation of the apparatus for the Oostenbrink direct cottonwool filter extraction method. Nematologica. 1963; 9: 106–110.

[24] Bernard EC, Handoo ZA, Powers TO, Donald PA, Heinz RD. *Vittatidera zeaphila* (Nematoda: Heteroderidae), a new genus and species of cyst nematode parasitic on corn (*Zea mays*). J Nematol. 2010; 42: 139–150. 22736850 PMC3380471

[25] Dong K, Opperman CH. Genetic analysis of parasitism in the soybean cyst nematode *Heterodera glycines*. Genetics. 1997; 146: 1311–1318. 9258676 10.1093/genetics/146.4.1311 PMC1208077

[26] Gardner M, Heinz R, Wang J, Mitchum MG. Genetics and adaption of soybean cyst nematode to broad spectrum resistance. G3. 2017; 10.1534/g3.116.035964 PMC5345713 28064187

[27] Skantar AM, Handoo ZA, Zanakis GN, Tzortzakakis EA. Molecular and morphological characterization of the corn cyst nematode, *Heterodera zeae*, from Greece. J Nematol. 2012; 44: 58–66. 23482617 PMC3593258

[28] Hoerauf A, Mand S, Adjei O, Fleischer B, Büttner DW. Depletion of wolbachia endobacteria in *Onchocerca volvulus* by doxycycline and microfilaridermia after ivermectin treatment. Lancet. 2001; 357: 1415–1416. 10.1016/S0140-6736(00)04581-5 11356444

