# Supplementary material for: Soybean cyst nematode culture collections and field populations from North Carolina and Missouri reveal high incidences of infection by viruses
Source: PLoS One. 2017 Jan 31;12(1):e0171514. doi: 10.1371/journal.pone.0171514 (PMC5283738; doi:10.1371/journal.pone.0171514)
Supplement: S1 Table — (DOCX) [file pone.0171514.s001.docx]

| Primer | Sequence [5’ to 3’] |
| --- | --- |
| ScNVSEQ | TCACGACTATGCTCTACACC |
| ScNV(-)SEQ | GCCTCGTCCTCCACATCAAG |
| ScPVSEQ | GACGCGCAAGATGGAAGACC |
| ScPV(-)SEQ | TTTCTTATGACCGACTGACC |
| ScRVSEQ | CAATGTGAAGTGCTACATC |
| ScRV(-)SEQ | ACGAGCCTCAAACCCGCAC |
| ScTVSEQ | CATGCCGGCCTCCACTCCGC |
| ScTV(-)SEQ | CCGGTGTAGCAGGGAGATCAGG |
| SbCNV-5SEQ | GCCCACCTATTTTACGAGC |
| SbCNV-5(-)SEQ | CTCAAGAGGTCAAACCGCG |
