# Supplementary material for: Soybean cyst nematode culture collections and field populations from North Carolina and Missouri reveal high incidences of infection by viruses
Source: PLoS One. 2017 Jan 31;12(1):e0171514. doi: 10.1371/journal.pone.0171514 (PMC5283738; doi:10.1371/journal.pone.0171514)
Supplement: S2 Table — The Ct value of each virus was normalized against the SCN internal control gene GAPDH. (DOCX) [file pone.0171514.s002.docx]

|  | Egg | | | | J2 | | | | | J3/J4 | | | | |  |  |
| --- | --- | --- | --- | --- | --- | --- | --- | --- | --- | --- | --- | --- | --- | --- | --- | --- |
|  | **^a^**AvgCt rep 1 | **^b^**AvgNAR rep 1 | Avg Ct rep 2 | Avg NAR rep 2 | | Avg Ct rep 1 | Avg NAR rep 1 | Avg Ct rep 2 | Avg NAR rep 2 | | Avg Ct rep 1 | Avg NAR rep 1 | Avg Ct rep 2 | Avg NAR rep 2 | |  |
| ScNV | 29.13 | 0.044 | 30.84 | 0.21 | | 33.33 | 0.12 | 26.71 | 0.043 | | 31.09 | 0.034 | 31.10 | 0.011 | |  |
| ScPV | 28.29 | 0.066 | 34.94 | 0.013 | | 34.33 | 0.050 | 28.30 | 0.013 | | 30.60 | 0.040 | 30.59 | 0.013 | |  |
| GAPDH | 21.62 | - | 25.31 | - | | 26.68 | - | 19.43 | - | | 23.01 | - | 21.47 | - | |  |
| ^a^ average cycle threshold (Ct; technical replicates repeated in triplicate)  ^b^ average normalized abundance ratio (NAR) | | | | | | | | | | | | | | | | |
