# Supplementary material for: Soybean cyst nematode culture collections and field populations from North Carolina and Missouri reveal high incidences of infection by viruses
Source: PLoS One. 2017 Jan 31;12(1):e0171514. doi: 10.1371/journal.pone.0171514 (PMC5283738; doi:10.1371/journal.pone.0171514)
Supplement: S3 Table — The Ct value of each virus was normalized against the SCN internal control gene GAPDH. (DOCX) [file pone.0171514.s003.docx]

|  | Egg | | | | J2 | | | | | J3/J4 | | | | |  |  |
| --- | --- | --- | --- | --- | --- | --- | --- | --- | --- | --- | --- | --- | --- | --- | --- | --- |
|  | **^a^**Avg Ct rep 1 | **^b^**AvgNAR rep 1 | Avg. Ct rep 2 | Avg. NAR rep 2 | | Avg. Ct rep 1 | Avg. NAR rep 1 | Avg. Ct rep 2 | Avg. NAR rep 2 | | Avg. Ct rep 1 | Avg. NAR rep 1 | Avg. Ct rep 2 | Avg. NAR rep 2 | |  |
| ScNV | 21.00 | 5.58 | 21.29 | 8.00 | | 29.57 | 16.57 | 21.63 | 4.19 | | 27.65 | 2.05 | 25.14 | 25.09 | |  |
| ScPV | 20.02 | 9.46 | 20.01 | 16.45 | | 29.04 | 20.14 | 19.91 | 11.50 | | 24.05 | 18.50 | 24.91 | 25.60 | |  |
| ScRV | 23.18 | 1.96 | 23.25 | 3.26 | | 32.17 | 5.13 | 23.14 | 2.27 | | 29.47 | 1.00 | 27.34 | 9.33 | |  |
| ScTV | 20.83 | 7.77 | 21.20 | 10.59 | | 29.59 | 22.44 | 21.13 | 7.24 | | 25.03 | 14.48 | 25.57 | 24.86 | |  |
| GAPDH | 21.04 | - | 21.79 | - | | 30.16 | - | 21.21 | - | | 25.58 | - | 26.79 | - | |  |
| ^a^ average cycle threshold (Ct; technical replicates repeated in triplicate)  ^b^ average normalized abundance ratio (NAR) | | | | | | | | | | | | | | | | |
