# Supplementary material for: Soybean cyst nematode culture collections and field populations from North Carolina and Missouri reveal high incidences of infection by viruses
Source: PLoS One. 2017 Jan 31;12(1):e0171514. doi: 10.1371/journal.pone.0171514 (PMC5283738; doi:10.1371/journal.pone.0171514)
Supplement: S4 Table — Data presented are the means of technical triplicates. Genomic and anti-genomic RNA was detected by initiating first strand cDNA synthesis with primers specific to each strand. Random primers were also used for cDNA synthesis as a control for both genomic and anti-genomic RNA. (DOCX) [file pone.0171514.s004.docx]

|  | Egg | | | J2 | | | J3/J4 | | |
| --- | --- | --- | --- | --- | --- | --- | --- | --- | --- |
|  | Genomic | Anti-genomic | Random | Genomic | Anti-genomic | Random | Genomic | Anti-genomic | Random |
| ScNV | 22.23 | 25.16 | 22.94 | 27.40 | 29.28 | 26.60 | 23.63 | 25.51 | 22.78 |
| ScPV | 24.59 | 31.04 | 25.30 | 31.40 | 33.70 | 31.09 | 25.49 | 31.51 | 25.34 |
| GAPDH | - | - | 23.34 | - | - | 28.50 | - | - | 23.92 |
| 18*S* | - | - | 9.04 | - | - | 12.85 | - | - | 10.63 |
