# Supplementary material for: Soybean cyst nematode culture collections and field populations from North Carolina and Missouri reveal high incidences of infection by viruses
Source: PLoS One. 2017 Jan 31;12(1):e0171514. doi: 10.1371/journal.pone.0171514 (PMC5283738; doi:10.1371/journal.pone.0171514)
Supplement: S5 Table — Data presented are the means of technical triplicates. Genomic and anti-genomic RNA was detected by initiating first strand cDNA synthesis with primers specific to each strand. Random primers were also used for cDNA synthesis as a control for both genomic and anti-genomic RNA. (DOCX) [file pone.0171514.s005.docx]

|  | Egg | | | J2 | | | J3/J4 | | |
| --- | --- | --- | --- | --- | --- | --- | --- | --- | --- |
|  | Genomic | Anti-genomic | Random | Genomic | Anti-genomic | Random | Genomic | Anti-genomic | Random |
| ScNV | 22.38 | 23.94 | 21.59 | 22.47 | 23.81 | 21.93 | 26.81 | 28.42 | 26.16 |
| ScPV | 18.43 | 22.01 | 18.30 | 18.72 | 21.47 | 18.60 | 23.35 | 27.17 | 23.19 |
| ScRV | 27.11 | 30.16 | 25.60 | 26.32 | 29.66 | 25.36 | 31.54 | 36.09 | 30.19 |
| ScTV | 21.52 | 23.04 | 20.62 | 21.34 | 22.89 | 20.87 | 25.99 | 28.55 | 25.25 |
| GAPDH | - | - | 21.79 | - | - | 21.21 | - | - | 26.79 |
| 18*S* | - | - | 8.93 | - | - | 8.15 | - | - | 11.57 |
