# Supplementary material for: Soybean cyst nematode culture collections and field populations from North Carolina and Missouri reveal high incidences of infection by viruses
Source: PLoS One. 2017 Jan 31;12(1):e0171514. doi: 10.1371/journal.pone.0171514 (PMC5283738; doi:10.1371/journal.pone.0171514)
Supplement: S6 Table — Data presented are the means of technical triplicates. SCN internal controls for relative quantification are HgFAR1 and GAPDH. (DOCX) [file pone.0171514.s006.docx]

| SCN Population | Internal Controls | | SCN Viruses | | | | |
| --- | --- | --- | --- | --- | --- | --- | --- |
|  | HgFAR1 | GAPDH | ScNV | ScPV | ScRV | ScTV | SbCNV-5 |
| LY1 | 26.19 | 26.88 | 25.68 | 28.09 | 30.74 | 27.09 | 22.35 |
| LY2 | 24.33 | 24.75 | 26.14 | 27.19 | 29.13 | 29.82 | 24.55 |
| MM1 | 26.42 | 26.56 | 28.35 | 29.20 | 33.03 | 28.78 | ND^b^ |
| MM2 | 20.74 | 20.65 | 22.35 | 22.24 | 27.45 | 23.53 | 32.79 |
| MM3 | 21.32 | 22.41 | 23.58 | 22.91 | 26.87 | 24.13 | ND |
| MM4 | 26.24 | 26.57 | 27.27 | 28.40 | 29.94 | 27.71 | ND |
| MM7 | 18.03 | 19.06 | 17.07 | 18.91 | 21.70 | 31.22 | ND |
| MM7/Williams82 | 26.41 | 26.23 | 26.47 | 28.07 | 31.54 | ND | ND |
| MM8 | 18.95 | 20.32 | 21.99 | 21.73 | 24.30 | 22.32 | ND |
| MM8/Williams82 | 20.46 | 21.22 | 22.68 | 22.97 | 27.57 | 23.36 | ND |
| MM8/Peking | 19.86 | 20.52 | 21.00 | 22.09 | 24.44 | 22.23 | ND |
| MM10 | 19.75 | 22.51 | 20.15 | 21.85 | 24.02 | 21.33 | ND |
| MM10/Williams82 | 18.45 | 21.76 | 18.58 | 20.28 | 22.29 | 20.21 | ND |
| MM16 | 18.37 | 18.88 | ND | ND | 32.37 | ND | ND |
| MM18 | 21.09 | 21.62 | 31.00 | 29.94 | ND | ND | ND |
| MM19 | 20.44 | 21.12 | 28.56 | ND | 33.58 | 30.81 | ND |
| MM21 | 24.19 | 24.44 | ND | ND | ND | ND | ND |
| MM23 | 27.25 | 27.60 | ND | ND | ND | ND | ND |
| MM24 | 29.23 | 29.06 | ND | ND | ND | ND | ND |
| OP20 | 21.88 | 22.26 | 23.21 | ND | ND | ND | ND |
| OP20^a^ | 19.91 | – | 29.02 | ND | ND | ND | ND |
| OP25 | 22.03 | 27.54 | 31.25 | 28.61 | ND | ND | 26.35 |
| OP25^a^ | 23.21 | – | 32.08 | 32.59 | ND | ND | ND |
| OP50 | 24.57 | 24.78 | 23.06 | ND | ND | ND | ND |
| OP50^a^ | 21.25 | – | 31.99 | 31.79 | ND | ND | ND |
| PA3 | 24.69 | 26.25 | 26.45 | 27.76 | 30.88 | 26.72 | ND |
| TN1 | 26.66 | 26.97 | 26.64 | ND | 29.82 | 28.59 | 23.72 |
| TN2/soybean | 24.36 | 25.17 | 25.81 | ND | ND | 34.15 | ND |
| TN2/tomato | 19.81 | 20.17 | 20.18 | ND | 25.57 | ND | 34.99 |
| TN6 | 25.18 | 26.29 | 34.39 | ND | ND | ND | 32.69 |
| TN7 (a) | 17.99 | 19.00 | 19.24 | 19.69 | 23.07 | 19.56 | 16.75 |
| TN7 (b) | 22.09 | 22.69 | 23.42 | 24.74 | 24.80 | 23.60 | 20.03 |
| TN8 (a) | 22.37 | 22.07 | 31.69 | ND | 20.70 | ND | 22.90 |
| TN8 (b) | 23.21 | 24.60 | ND | ND | 34.57 | ND | 21.98 |
| TN12 | 26.79 | 27.94 | 27.18 | 30.58 | 31.51 | 28.48 | ND |
| TN13 | 21.33 | 22.13 | 21.17 | ND | 24.66 | 22.51 | 20.18 |
| TN14 | 26.66 | 27.26 | 27.04 | ND | 32.47 | ND | ND |
| TN15 | 26.82 | 26.23 | 26.87 | ND | 31.52 | 28.01 | ND |
| TN19 | 26.01 | 27.86 | 27.64 | 33.22 | 31.72 | 28.93 | ND |
| TN20 | 26.01 | 26.26 | ND | ND | 34.32 | ND | 30.89 |
| TN21 | 18.61 | 19.60 | 18.87 | 21.00 | 23.18 | 21.09 | ND |
| TN22 | 18.38 | 18.53 | 18.07 | 19.63 | 27.45 | 19.76 | 30.93 |
| VL1 | 23.98 | 25.04 | 34.69 | ND | 30.37 | 25.42 | ND |
| ^a^ Samples are maintained in NCSU greenhouses; all other samples are from MU greenhouses  ^b^ virus not detected (ND) | | | | | | | |
