# Supplementary material for: Soybean cyst nematode culture collections and field populations from North Carolina and Missouri reveal high incidences of infection by viruses
Source: PLoS One. 2017 Jan 31;12(1):e0171514. doi: 10.1371/journal.pone.0171514 (PMC5283738; doi:10.1371/journal.pone.0171514)
Supplement: S7 Table — Data presented are the means of technical triplicates. The SCN internal control HgFAR1 is used for relative quantification of virus abundance. (DOCX) [file pone.0171514.s007.docx]

| SCN Population | Internal Controls | | SCN Viruses | | | | |
| --- | --- | --- | --- | --- | --- | --- | --- |
|  | HgFAR1 | GAPDH | ScNV | ScPV | ScRV | ScTV | SbCNV-5 |
| Beaufort | 23.86 | 25.05 | 34.96 | 34.39 | ND^b^ | ND | ND |
| Bertie | 25.75 | 26.99 | ND | 33.88 | ND | ND | ND |
| Bladen | 25.83 | 27.50 | 30.32 | ND | ND | ND | ND |
| Columbus | 18.78 | – | 23.76 | 27.54 | ND | ND | 34.47 |
| Craven | 28.61 | 27.78 | 30.78 | ND | ND | ND | ND |
| Duplin | 19.38 | – | 27.62 | ND | 30.19 | ND | ND |
| Edgecombe | 29.47 | – | ND | 33.95 | ND | ND | 29.52 |
| Gates | 20.74 | – | 25.72 | ND | ND | ND | ND |
| Greene | 24.12 | 25.51 | 32.42 | 33.69 | ND | ND | ND |
| Guilford | 22.99 | 23.50 | 28.93 | 31.42 | 34.50 | ND | ND |
| Hertford | 20.96 | – | 25.53 | 32.24 | ND | ND | ND |
| Jones | 22.57 | – | 25.76 | ND | ND | ND | ND |
| Lee | 23.48 | – | ND | ND | ND | ND | ND |
| Onslow | 24.53 | 21.06 | 28.13 | 32.40 | ND | ND | ND |
| Pamlico | 21.52 | – | 23.49 | 28.25 | ND | ND | 34.23 |
| Person | 20.76 | – | 30.10 | 29.76 | ND | ND | ND |
| Robeson | 24.18 | 25.31 | 25.75 | 33.80 | 31.79 | ND | ND |
| Tyrell | 23.01 | 24.96 | ND | 32.93 | ND | ND | ND |
| Wake | 25.18 | 25.61 | 30.72 | 34.07 | ND | ND | ND |
| Washington | 26.92 | – | 32.51 | ND | ND | ND | ND |
| Barton**^a^** | 20.22 | 17.51 | 31.75 | ND | ND | ND | ND |
| Dunklin 1**^a^** | 21.60 | 22.53 | ND | ND | ND | ND | ND |
| Dunklin 2**^a^** | 24.35 | 25.19 | ND | ND | ND | ND | ND |
| Scott 1**^a^** | 24.99 | 25.66 | ND | ND | ND | ND | ND |
| Scott 2**^a^** | 24.03 | 24.62 | ND | ND | ND | ND | ND |
| ^a^ Samples were collected from infested fields in Missouri; all other samples are from North Carolina fields  ^b^ virus not detected (ND) | | | | | | | |
